# Metabolic engineering of *Escherichia coli* for the synthesis of polyhydroxyalkanoates using acetate as a main carbon source

**DOI:** 10.1186/s12934-018-0949-0

**Published:** 2018-07-03

**Authors:** Jing Chen, Wei Li, Zhao-Zhou Zhang, Tian-Wei Tan, Zheng-Jun Li

**Affiliations:** 1 Beijing Advanced Innovation Center for Soft Matter Science and Engineering, Beijing University of Chemical Technology, Mailbox 53, No. 15 Beisanhuan Donglu, Chaoyang District, Beijing, 100029 China; 2 Beijing Key Laboratory of Bioprocess, College of Life Science and Technology, Beijing University of Chemical Technology, Beijing, 100029 China

**Keywords:** Acetate, *Escherichia coli*, Poly-3-hydroxybutyrate, Poly(3-hydroxybutyrate-*co*-4-hydroxybutyrate), Poly(3-hydroxybutyrate-*co*-3-hydroxyvalerate)

## Abstract

**Background:**

High production cost of bioplastics polyhydroxyalkanoates (PHA) is a major obstacle to replace traditional petro-based plastics. To address the challenges, strategies towards upstream metabolic engineering and downstream fermentation optimizations have been continuously pursued. Given that the feedstocks especially carbon sources account up to a large portion of the production cost, it is of great importance to explore low cost substrates to manufacture PHA economically.

**Results:**

*Escherichia coli* was metabolically engineered to synthesize poly-3-hydroxybutyrate (P3HB), poly(3-hydroxybutyrate-*co*-4-hydroxybutyrate) (P3HB4HB), and poly(3-hydroxybutyrate-*co*-3-hydroxyvalerate) (PHBV) using acetate as a main carbon source. Overexpression of phosphotransacetylase/acetate kinase pathway was shown to be an effective strategy for improving acetate assimilation and biopolymer production. The recombinant strain overexpressing phosphotransacetylase/acetate kinase and P3HB synthesis operon produced 1.27 g/L P3HB when grown on minimal medium supplemented with 10 g/L yeast extract and 5 g/L acetate in shake flask cultures. Further introduction succinate semialdehyde dehydrogenase, 4-hydroxybutyrate dehydrogenase, and CoA transferase lead to the accumulation of P3HB4HB, reaching a titer of 1.71 g/L with a 4-hydroxybutyrate monomer content of 5.79 mol%. When 1 g/L of α-ketoglutarate or citrate was added to the medium, P3HB4HB titer increased to 1.99 and 2.15 g/L, respectively. To achieve PHBV synthesis, acetate and propionate were simultaneously supplied and propionyl-CoA transferase was overexpressed to provide 3-hydroxyvalerate precursor. The resulting strain produced 0.33 g/L PHBV with a 3-hydroxyvalerate monomer content of 6.58 mol%. Further overexpression of propionate permease improved PHBV titer and 3-hydroxyvalerate monomer content to 1.09 g/L and 10.37 mol%, respectively.

**Conclusions:**

The application of acetate as carbon source for microbial fermentation could reduce the consumption of food and agro-based renewable bioresources for biorefineries. Our proposed metabolic engineering strategies illustrate the feasibility for producing polyhydroxyalkanoates using acetate as a main carbon source. Overall, as an abundant and renewable resource, acetate would be developed into a cost-effective feedstock to achieve low cost production of chemicals, materials, and biofuels.

**Electronic supplementary material:**

The online version of this article (10.1186/s12934-018-0949-0) contains supplementary material, which is available to authorized users.

## Background

Food and agro-based renewable bioresources, such as xylose [1], glucose [2], sucrose [3], and starch [4], are most widely used carbon feedstocks for the microbial production of polymers, biofuels and building block chemicals. Polyhydroxyalkanoates (PHA), the promising biodegradable polyesters which are produced by an extensive variety of microorganisms for carbon and energy storage purposes, are also synthesized from glucose as a main carbon source [5, 6]. Although considerable efforts have been devoted to decreasing the production cost of PHA to make it economically competitive, the present price of PHA is still not feasible to replace traditional petro-based plastics [7]. It is believed that the feedstocks especially carbon source accounts up to a large portion of the production cost. Therefore, it is important to develop low cost substrates to make PHA economically competitive [8]. In addition, the global food shortage and the price increases of agricultural products have made it difficult to rely on food-based materials as fermentation feedstocks, in addition to the ethical considerations involved with doing so. Consequently, the development of non-food based substrates, such as methanol [9], acetate [10], and syngas [11], is quickly becoming one of the most important research areas of industrial biotechnology.

Acetate is the second simplest carboxylic acid, and many microorganisms can utilize acetate as alternative carbon source for cell growth. The acetate assimilation species include *Escherichia coli* [12], *Cryptococcus curvatus* [13], *Clostridium* sp. [14], *Halomonas boliviensis* [15], and so on. Recent studies have demonstrated that acetate can be produced from low cost substrates via biochemical processes. For example, acetate is a major fermentation product during anaerobic digestion of organic wastes and syngas production by acetogenic bacteria *Moorella thermoacetica* from carbon dioxide [16, 17]. In addition, acetate is also widely existed as a byproduct from hydrolysis or pyrolysis of lignocellulosic biomass [18, 19]. In this regard, compared with glucose, acetate would be a promising cost-effective carbon source suitable for microbial fermentation.

Oleaginous yeasts including *C*. *curvatus* [20] and *Yarrowia lipolytica* [21] have been reported to utilize waste acetate as a main carbon source to accumulate lipids. Although acetate is a notorious cell growth inhibitor for *E. coli* fermentation, recombinant *E. coli* strains were constructed to use acetate for the production of free fatty acids [22] and succinate [23]. Therefore, it is also quite interesting to investigate the possibility of producing PHA polymers from acetate.

**Fig. 1:**
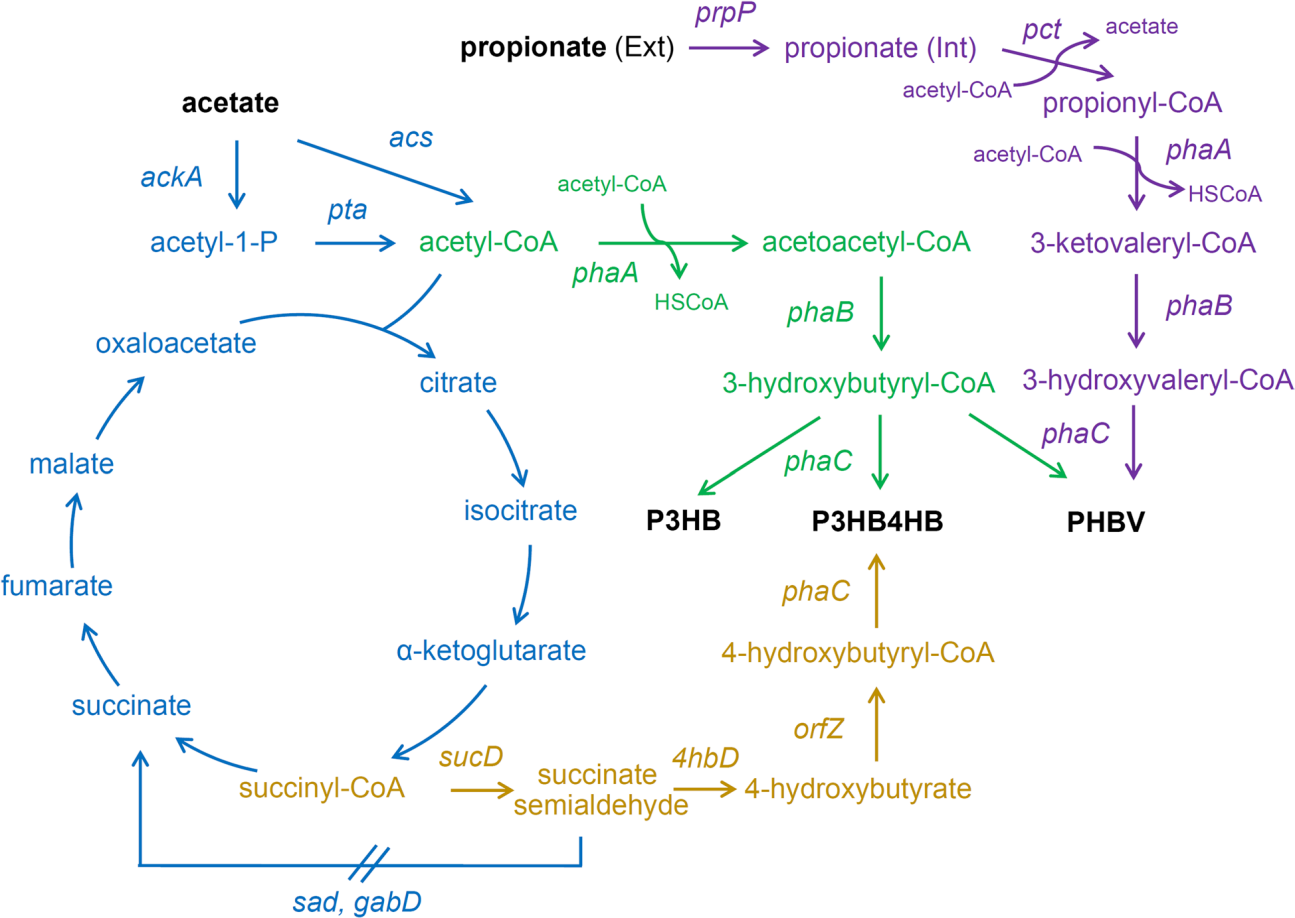
P3HB, P3HB4HB, and PHBV synthesis pathway in engineered *Escherichia coli.* Genes: *acs*, acetyl-CoA synthetase; *pta,* phosphotransacetylase; *ackA*, acetate kinase; *phaA,* β-ketothiolase; *phaB*, acetoacetyl-CoA reductase; *phaC*, PHA synthase; *sucD*, succinate semialdehyde dehydrogenase; *4hbD*, 4-hydroxybutyrate dehydrogenase; *orfZ*, 4-hydroxybutyryl-CoA:CoA transferase; *sad* and *gabD*, succinate semialdehyde dehydrogenase of *E. coli*; *pct*, propionyl-CoA transferase; *prpP*, propionate permease. The reactions and intermediates shown in green are associated with 3HB precursor biosynthesis, those in gold with 4HB precursor biosynthesis, those in purple with 3HV precursor biosynthesis, and those in blue with acetate assimilation and TCA cycle

In this study, the metabolic pathways of *E. coli* were engineered to produce poly-3-hydroxybutyrate (P3HB), poly(3-hydroxybutyrate-*co*-4-hydroxybutyrate) (P3HB4HB), and poly(3-hydroxybutyrate-*co*-3-hydroxyvalerate) (PHBV) using acetate as a main carbon source (Fig. 1). We compared the effects of overexpressing phosphotransacetylase/acetate kinase and AMP-forming acetyl-CoA synthetase on acetate assimilation and P3HB production. In addition, succinate semialdehyde dehydrogenase, 4-hydroxybutyrate dehydrogenase, and CoA transferase was overexpressed to construct P3HB4HB accumulation pathway. Propionyl-CoA transferase and propionate permease were introduced to provide 3-hydroxyvalerate precursor from propionate, which helped to achieve PHBV synthesis. Our results demonstrate that P3HB, P3HB4HB, and PHBV can be effectively synthesized using acetate as a main carbon source, opening new approaches for the utilization of this cheap carbon source for the production of various high value-added biomaterials.

## Methods

### Bacterial strains and culture medium

*Escherichia coli* JM109 was employed as the host for plasmid construction and P3HB and PHBV production studies, while its succinate semialdehyde dehydrogenase mutant JM109SG [24] was used as the host for P3HB4HB production.

For construction of recombinants and plasmid amplification, *E. coli* strains were cultivated in Luria–Bertani (LB) medium at 37 °C and 200 rpm. LB medium contained 5 g/L yeast extract, 10 g/L Bacto tryptone, and 10 g/L NaCl. To maintain plasmid stability, 100 μg/mL ampicillin and/or 50 μg/mL kanamycin was added to the media when required.

### Plasmid construction

Strains and plasmids used in this study are listed in Table 1. Oligonucleotides used in this study are listed in Additional file 1: Table S1. Standard molecular cloning procedures or manufacturers’ instructions were followed for plasmid construction. Q5^®^ High-Fidelity DNA Polymerase from New England Biolabs (Ipswich, MA, US) was employed for PCR reactions. Plasmid isolation and DNA purification kits were purchased from Biomed (Beijing, China). Restriction enzymes and DNA modifying enzymes were purchased from New England Biolabs.

**Table 1:** Strains and plasmids used in this study

Name	Relevant characteristics	References
*E. coli* strains		
JM109	Wild type	[24]
JM109SG	JM109 *Δsad ΔgabD*	[24]
JM109 (pBHR68)	P3HB producing strain	This study
JM109 (pBHR68 + pBBR1MCS-2)	P3HB producing strain	This study
JM109 (pBHR68 + pMCS-pta-ackA)	P3HB producing strain	This study
JM109 (pBHR68 + pMCS-acs)	P3HB producing strain	This study
JM109SG (p68orfZ + pMCSH5)	P3HB4HB producing strain	This study
JM109SG (p68orfZ + pMCSH5-pta-ackA)	P3HB4HB producing strain	This study
JM109 (p68-pct-CAB + pBBR1MCS-2)	PHBV producing strain	This study
JM109 (p68-pct-CAB + pMCS-pta-ackA)	PHBV producing strain	This study
JM109 (p68-pct-CAB + pMCS-pta-ackA-prpP)	PHBV producing strain	This study
Plasmids		
pBHR68	*phaCAB* expression plasmid	[25]
p68orfZ	*orfZ* inserted into pBHR68	[24]
pEn	Expression vector, P_pdc_ promoter	[24]
pBBR1MCS-2	Broad-host-range vector	[26]
pMCSH5	P_pdc_-*sucD*-*4hbD* inserted into pBBR1MCS-2	[24]
p68-pct-CAB	*pct* and *phaCAB* expression plasmid	[27]
pEn-pta-ackA	*pta*-*ackA* inserted into pEn	This study
pEn-acs	*acs* inserted into pEn	This study
pEn-prpP	*prpP* inserted into pEn	This study
pMCS-pta-ackA	P_pdc_-*pta*-*ackA* inserted into pBBR1MCS-2	This study
pMCS-acs	P_pdc_-*acs* inserted into pBBR1MCS-2	This study
pMCSH5-pta-ackA	P_pdc_-*pta*-*ackA* inserted into pMCSH5	This study
pMCS-pta-ackA-prpP	P_pdc_-*pta*-*ackA*-P_pdc_-*prpP* inserted into pBBR1MCS-2	This study

The *pta*-*ackA* encoding phosphotransacetylase/acetate kinase and *acs* encoding acetyl-CoA synthetase were amplified from *E. coli* genomic DNA using primers pa1F/pa1R and acsF/acsR, respectively. The PCR products were purified and digested with *Hind*III/*Xba*I and inserted into the corresponding restriction sites of pEn to construct pEn-pta-ackA and pEn-acs, respectively. Moreover, P_pdc_ promoter and gene coding sequence were removed from pEn-derived plasmids and subcloned into kanamycin resistance carrying broad-host-range cloning vector pBBR1MCS-2 to generate pMCS-pta-ackA and pMCS-acs, respectively. Primers pa2F/pa2R were designed to amply *pta*-*ackA* from pEn-pta-ackA. The PCR product was digested with *Nhe*I*/Xho*I and cloned into the *sucD*-*4hbD* expression vector pMCSH5 cut with the same enzymes to form pMCSH5-pta-ackA. Primers prpPF/prpPR were designed to amply *prpP* encoding propionate permease from *Ralstonia eutropha* H16 genomic DNA. The PCR product was digested with *Bam*HI*/Hind*III and cloned into pEn to generate pEn-prpP. Subsequently, P_pdc_-prpP was excised from pEn-prpP using *Spe*I*/Xho*I and cloned into pMCS-pta-ackA digested with *Nhe*I*/Xho*I to generate pMCS-pta-ackA-prpP.

### Growth conditions for biopolymer production

To prepare the seed cultures, 10 μL of glycerol stock was inoculated into 20 mL of LB medium and cultivated at 37 °C and 200 rpm for 12 h. For biopolymer producing shake flask studies, 4% (v/v) of the seed culture was used to inoculate into 500 mL shake flasks containing 50 mL of medium and cultured at 37 °C and 200 rpm for 48 h.

The plasmids including pBBR1MCS-2, pMCS-pta-ackA, and pMCS-acs, each in combination with *phaCAB* expression plasmid pBHR68, were co-transformed into *E. coli* JM109 to generate P3HB producing strains. Three different kinds of fermentation media were employed for P3HB production: LB, terrific broth (TB), and minimal medium (MM). TB medium contained 24 g/L yeast extract, 12 g/L Bacto tryptone, 2.31 g/L KH_2_PO_4_, and 12.54 g/L K_2_HPO_4_. MM medium comprised 2 g/L NH_4_Cl, 5.0 g/L (NH_4_)_2_SO_4_, 6.0 g/L KH_2_PO_4_, 8.4 g/L MOPS, 0.5 g/L NaCl, 0.24 g/L MgSO_4_, 0.002 g/L Na_2_MoO_4_ and 1 mL/L trace element solution. The trace elements solution contained (g/L): FeCl_2_·4H_2_O 3.6, CaCl_2_·2H_2_O 5.0, MnCl_2_·2H_2_O 1.3, CuCl_2_·2H_2_O 0.38, CoCl_2_·6H_2_O 0.5, ZnCl_2_ 0.94, H_3_BO_3_ 0.0311, Na_2_EDTA·2H_2_O 0.4, and thiamine-HCl 1.01. When necessary, MM medium was supplemented with a certain amount of yeast extract.

Plasmids pMCSH5 and pMCSH5-pta-ackA, each together with *phaCAB*-*orfZ* expression plasmid p68orfZ, were co-transformed into *E. coli* JM109SG to generate P3HB4HB producing strains. The resulting recombinants were cultivated in MM medium supplemented with 10 g/L yeast extract and 5 g/L acetate for P3HB4HB accumulation. When necessary, α-ketoglutarate or citrate was added as a second carbon source at a concentration of 1 g/L.

Plasmids pBBR1MCS-2, pMCS-pta-ackA, and pMCS-pta-ackA-prpP, each together with *pct*-*phaCAB* expression plasmid p68-pct-CAB, were co-transformed into *E. coli* JM109 to generate PHBV producing strains. The resulting recombinants were cultivated in MM medium supplemented with 10 g/L yeast extract, 5 g/L acetate, and 1.5 g/L propionate for PHBV production.

### Analytical methods

Bacterial cells were harvested by centrifugation at 8000*g* for 10 min. The cell pellets were washed twice with distilled water and then lyophilized for 12 h for cell dry weight (CDW) measurement. Lyophilized cells were subjected to methanolysis at 100 °C for 4 h in the presence of 3% (v/v) H_2_SO_4_. Intracellular polymer content (wt%) and composition was analyzed using gas chromatography (GC) (Hewlett-Packard 6890) equipped with a capillary column HP-5 (30 m, 0.25 mm) and a flame-ionization detector. PHBV and γ-butyrolactone purchased from Sigma-Aldrich (St. Louis, MO, USA) were used as analysis standards. Residual cell weight (rCDW) is defined as non-PHA cell mass. rCDW in each sample was determined by substracting the PHA concentration measured by GC analysis from CDW.

For acetate detection, the supernatant of culture broth was filtered through a 0.2-μm syringe filter and stored chilled for high-performance liquid chromatography (HPLC) analysis equipped with an ion exchange column (Aminex^®^ HPX-87H, BioRad) and a refractive index detector (RI-150, Thermo Spectra System, USA). A mobile phase of 7 mM H_2_SO_4_ at a 0.6 mL/min flow rate was used.

## Results and discussion

### Pathway construction for P3HB synthesis from acetate

Over the past few decades, researchers have mainly been studying the “acetate switch” with the aim to enable microorganisms to efficiently consume acetate that is produced by their own metabolism to eliminate the toxicity of acetate to cell growth [28, 29]. Nonetheless, acetate could be a potential cost-effective feedstock for synthesis of value-added chemicals. Recently, the feasibility of converting acetate to poly-3-hydroxybutyrate (P3HB) and succinate was demonstrated in *Y. lipolytica* [30] and *E. coli* [23], respectively. Moreover, co-production of hydrogen and P3HB with engineered *E. coli* on glucose and acetate under anaerobic condition was reported. The addition of acetate to the culture as part of carbon source significantly increased P3HB production, yet the P3HB titer was still below 0.04 g/L [31].

**Fig. 2:**
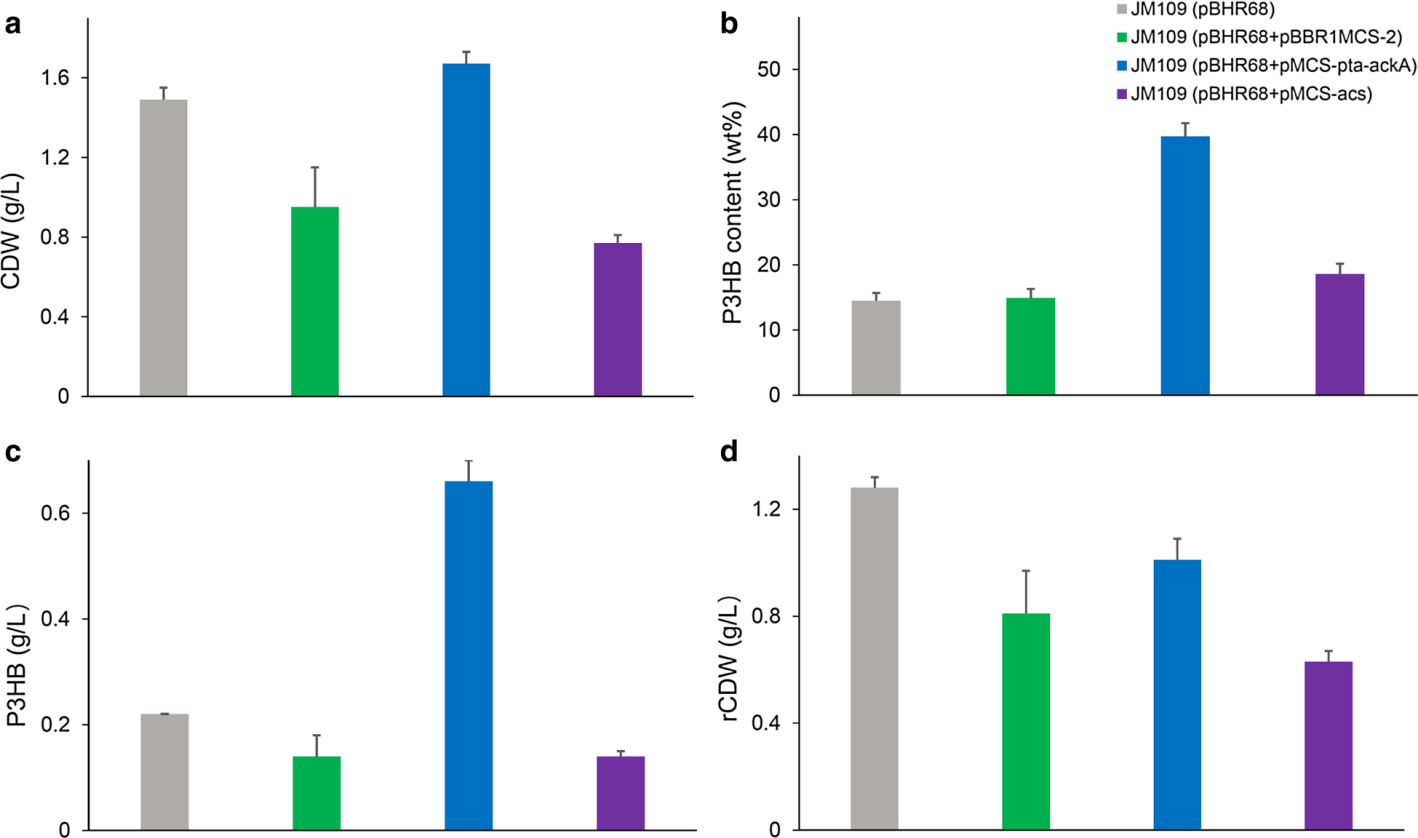
Effects of the overexpression of *pta*-*ackA* and *acs* on cell growth and P3HB production. *E. coli* recombinants harboring different plasmids were cultivated in MM medium supplemented with 5 g/L acetate and 2 g/L yeast extract at 37 °C for 48 h. CDW (**a**), P3HB content (**b**), P3HB titer (**c**), and rCDW (**d**) were measured. The columns represent the averages of triplicate experiments, and the error bars represent standard deviation

To investigate the possibility of P3HB production using acetate as a main carbon source in *E. coli*, P3HB synthesis operon *phaCAB* carrying plasmid pBHR68 was transformed into *E. coli* JM109, and the recombinant strain was inoculated into MM medium supplemented with 2 g/L yeast extract and 5 g/L acetate (Fig. 2). After 48 h shake flask cultivation, *E. coli* JM109 (pBHR68) consumed all the acetate and yielded 1.49 g/L CDW with 0.22 g/L P3HB accumulation, which indicated that P3HB could be produced from acetate, yet the P3HB titer was much lower than that obtained from glucose [32]. Therefore, further metabolic engineering strategies were applied to improve P3HB accumulation from acetate.

### Pathway engineering for improved P3HB accumulation

In *E. coli*, there are two potential routes for the generation of acetyl-CoA from acetate: phosphotransacetylase/acetate kinase (*pta*-*ackA* pathway) and AMP-forming acetyl-CoA synthetase (*acs* pathway) (Fig. 1). These two pathways were augmented through the overexpression of *pta*-*ackA* and *acs* genes, respectively. Shake flask cultivations in acetate containing MM medium showed that JM109 (pBHR68 + pMCS-pta-ackA) consumed 5 g/L acetate and produced 0.66 g/L P3HB. The P3HB production titer of was roughly four times of that produced by control strain JM109 (pBHR68 + pBBR1MCS-2). By contrast, the overexpression of *acs* gene has little effect on improving P3HB accumulation (Fig. 2). The activation of each molecule of acetate for acetyl-CoA synthesis in *acs* pathway requires two molecule of ATP, while *pta*-*ackA* pathway consumes only one molecule of ATP for acetyl-CoA synthesis [22, 33]. It was reported also that the growth of *E. coli* stains on low concentrations of acetate depends on *acs* pathway, while growth on high concentrations requires *pta*-*ackA* pathway [28, 29]. Therefore, the engineering of the *pta*-*ackA* pathway to strengthen acetate assimilation was considered to be an effective strategy for improving P3HB production in recombinant *E. coli*.

### Optimizing culture conditions to increase P3HB production

**Fig. 3:**
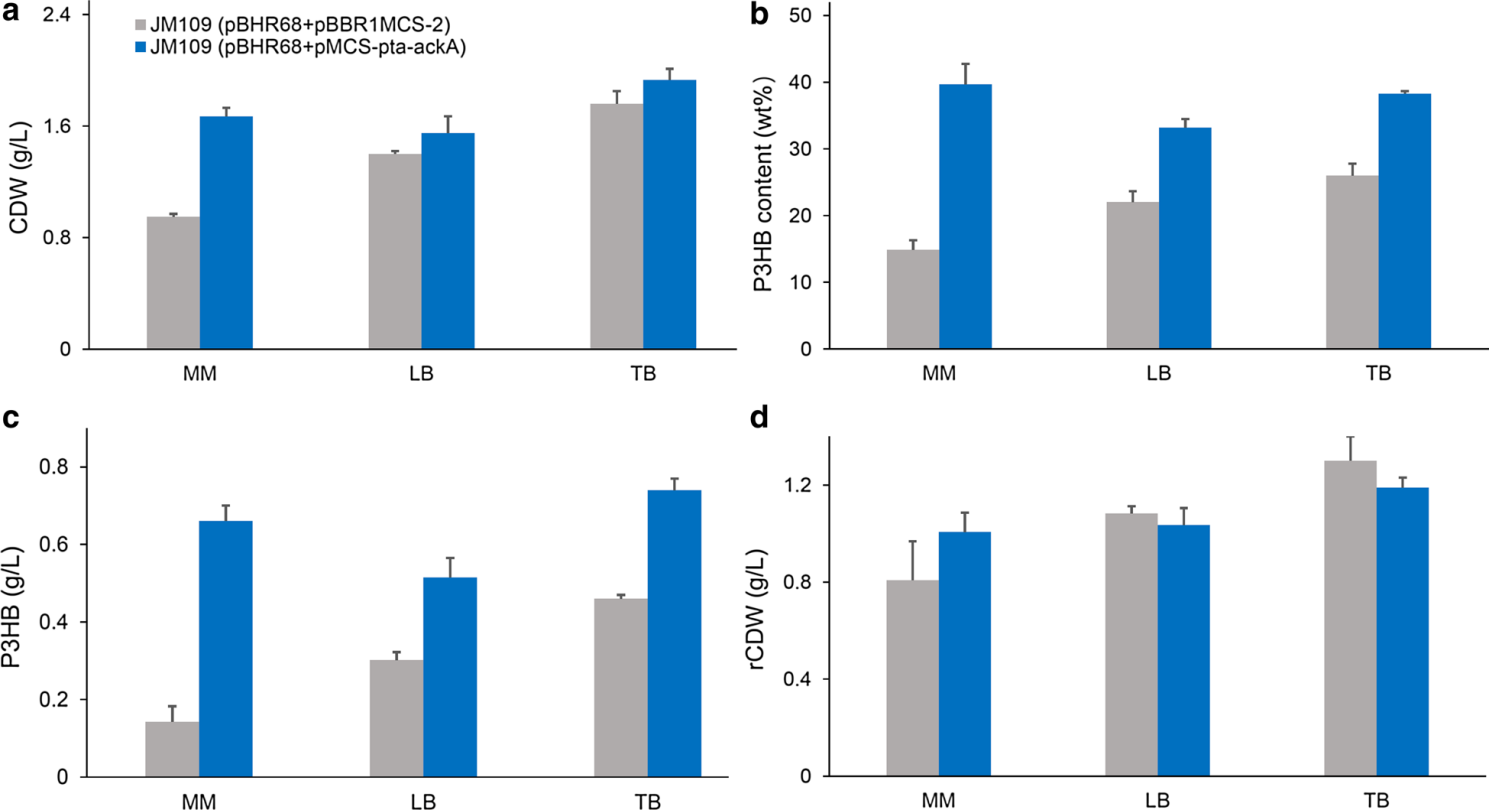
Effects of medium composition on cell growth and P3HB production. *E. coli* recombinants harboring different plasmids were cultivated in MM, LB, or TB medium supplemented with 5 g/L acetate at 37 °C for 48 h. MM medium contained 2 g/L yeast extract. CDW (**a**), P3HB content (**b**), P3HB titer (**c**), and rCDW (**d**) were measured. The columns represent the averages of triplicate experiments, and the error bars represent standard deviation

The engineered *E. coli* JM109 (pBHR68 + pMCS-pta-ackA) and control strain JM109 (pBHR68 + pBBR1MCS-2) were cultivated in shake flasks with LB, TB, and MM medium to evaluate the effects of medium composition on cell growth and P3HB accumulation (Fig. 3). Initial acetate concentration was 5 g/L and MM medium was supplemented with 2 g/L yeast extract. After 48 h cultivation, no acetate was left in the medium for all groups. The recombinant strain harboring extra *pta*-*ackA* genes produced more CDW and P3HB in the three different kinds of culture media, further demonstrated that *pta*-*ackA* overexpression was effective for improving P3HB production from acetate (Fig. 3). With MM medium, JM109 (pBHR68 + pMCS-pta-ackA) produced 1.67 g/L CDW, containing 0.66 g/L P3HB, while the use of LB and TB medium resulted in 1.55 g/L CDW with 0.52 g/L P3HB, and 1.93 g/L CDW with 0.74 g/L P3HB, respectively. TB medium helped to obtain higher CDW and P3HB titer than those achieved in MM and LB medium. Among three culture conditions, MM medium possesses lowest cost and resulted in acceptable P3HB titer. Therefore, it was considered to be a favorable medium for the production of P3HB from acetate.

**Fig. 4:**
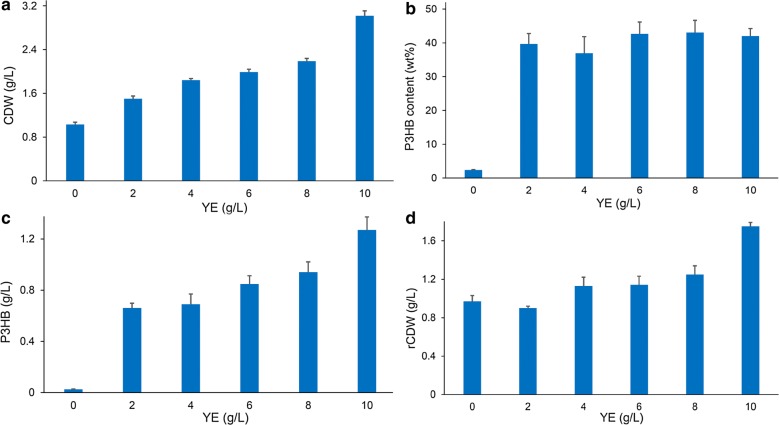
Effect of yeast extract concentration on cell growth and P3HB production. *E. coli* JM109 (pBHR68 + pMCS-pta-ackA) was cultivated in MM medium supplemented with 5 g/L acetate at 37 °C for 48 h. Different concentration (0, 2, 4, 6, 8, 10 g/L) of yeast extract (YE) was added to the culture. CDW (**a**), P3HB content (**b**), P3HB titer (**c**), and rCDW (**d**) were measured. The columns represent the averages of triplicate experiments, and the error bars represent standard deviation

Next, different concentration (0, 2, 4, 6, 8, 10 g/L) of yeast extract were added to MM medium to study its potential for enhancing cell growth and P3HB accumulation (Fig. 4). When yeast extract was not provided, CDW of *E. coli* JM109 (pBHR68 + pMCS-pta-ackA) reached 1.03 g/L, and P3HB concentration was only 0.02 g/L. The CDW and P3HB accumulation increased gradually with the increase of yeast extract concentration, although the intracellular P3HB content did not change obviously. The addition of 10 g/L yeast extract yielded 3.02 g/L CDW, containing 1.27 g/L P3HB, which was the highest P3HB production titer obtained in this study. The production yield was 0.25 g P3HB/g acetate, which was 35% of the maximal theoretical yield. It has been shown that yeast extract contributes a large amount of amino acids for biomass growth, thus the addition of complex nitrogen sources such as yeast extract to defined medium saved NADPH for amino acids synthesis and increased intracellular NADPH level as well as NADPH/NADP ratio [34]. P3HB biosynthesis is a NADPH-dependent pathway and high NADPH level and availability were considered to be of great importance for the efficient synthesis of PHB in recombinant *E. coli* [32]. Therefore, we speculate that the addition of yeast extract favored cell growth and intracellular NADPH availability which in turn led to high biomass and P3HB production.

We next cultivated *E. coli* JM109 (pBHR68 + pMCS-pta-ackA) using MM medium supplemented with 10 g/L yeast extract and different concentration (0, 1, 2, 3, 4, 5 g/L) of acetate to evaluate the effect of acetate concentration on P3HB accumulation. As shown in Additional file 1: Figure S1, when acetate was not provided, the CDW reached 1.15 g/L and the intracellular P3HB content was only 3.23 wt%. With the increase of acetate concentration, the CDW, P3HB content, and P3HB titer all increased gradually. The addition of 5 g/L acetate led to the highest P3HB content of 42.02 wt%. Although yeast extract was provided at a relatively high level, P3HB synthesis was still limited by acetate addition. In contrast, the results of shake flask cultures using 5 g/L acetate and different amounts of yeast extract showed that the P3HB content did not change obviously when yeast extract concentration increased from 2 to 10 g/L (Fig. 4). These results demonstrated that the carbon source existed in yeast extract was not able to support effective P3HB synthesis, and acetate was the main carbon source to synthesize P3HB.

### Production of P3HB4HB from acetate

Poly(3-hydroxybutyrate-*co*-4-hydroxybutyrate) (P3HB4HB) exhibits favorable biodegradability and a wide range of physical properties ranging from highly crystalline plastic to elastic rubber, thus it is considered to be one of the most promising PHA materials. The synthesis of P3HB4HB copolymer from structural unrelated carbon sources such as glucose in *E. coli* has been reported [24, 35, 36]. Nevertheless, in terms of substrate cost, acetate is a promising carbon source for microbial fermentations. Therefore, we aimed to developed an engineered *E. coli* that can produce P3HB4HB from acetate as carbon source.

**Fig. 5:**
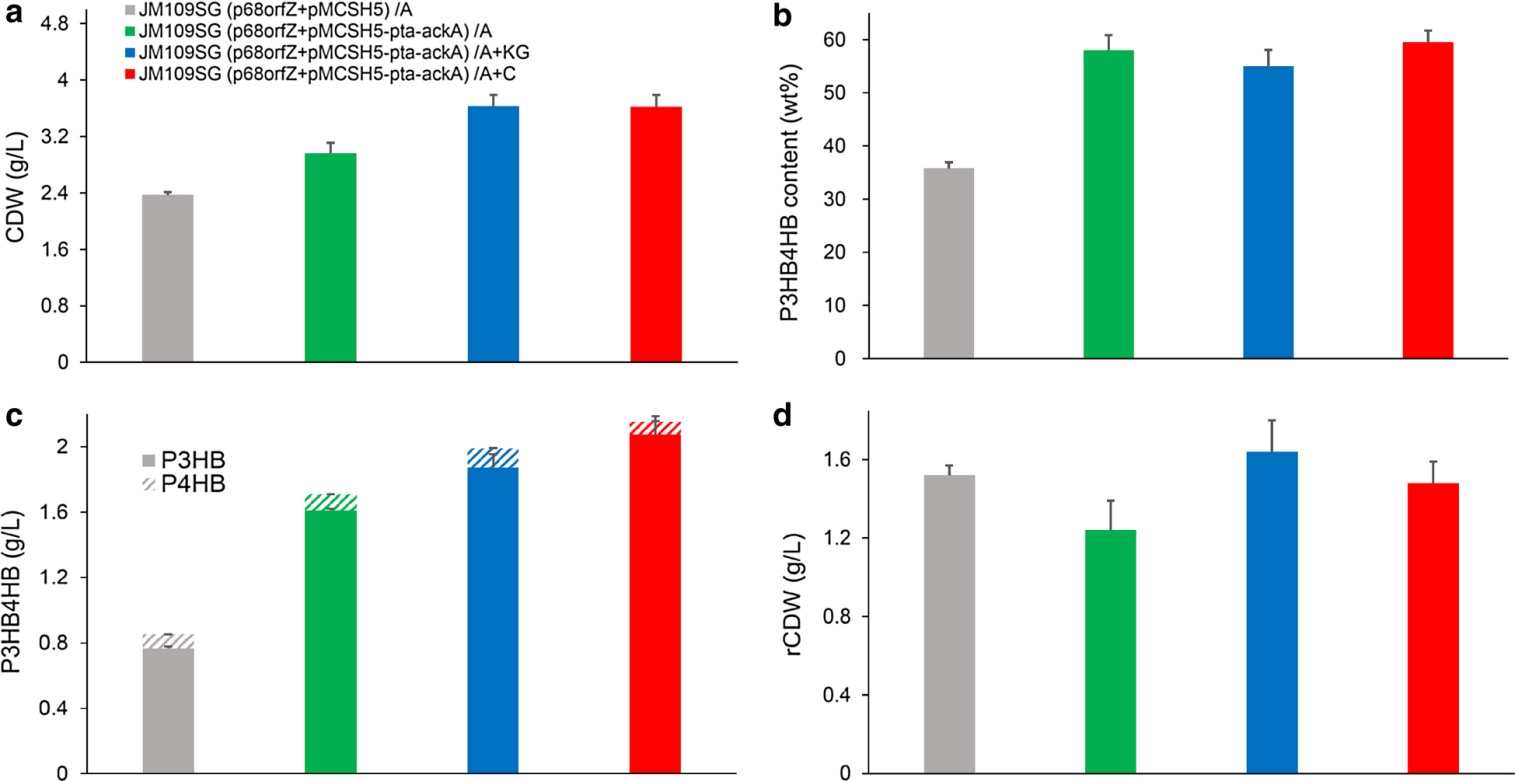
P3HB4HB production by *E. coli* strains grown in shake flasks. *E. coli* recombinants harboring different plasmids were cultivated in MM medium supplemented with 10 g/L yeast extract and different carbon sources at 37 °C for 48 h. Initial acetate (A) concentration was 5 g/L. α-ketoglutarate (KG) or citrate (C) was added as assistant carbon source at a concentration of 1 g/L. CDW (**a**), P3HB4HB content (**b**), P3HB4HB titer (**c**), and rCDW (**d**) were measured. The columns represent the averages of triplicate experiments, and the error bars represent standard deviation

The genes involved in succinate degradation pathway of *Clostridium kluyveri*, including *sucD*, *4hbD*, and *orfZ* [37], was combined with P3HB synthesis operon *phaCAB* to construct P3HB4HB producing pathway. *E. coli* native succinate semialdehyde dehydrogenase genes *sad* and *gabD* were both deleted for eliminating succinate formation from succinate semialdehyde (Fig. 1). The resulting strain JM109SG (p68orfZ + pMCSH5) was able to achieve 2.37 g/L CDW containing 35.79 wt% P(3HB-*co*-9.48 mol% 4HB) when grown in mineral medium supplemented with 10 g/L yeast extract and 5 g/L acetate (Fig. 5). In addition, the *pta*-*ackA* genes were overexpressed to strengthen acetate assimilation, and the recombinant JM109SG (p68orfZ + pMCSH5-pta-ackA) yielded 2.96 g/L CDW, containing 58.01 wt% P(3HB-*co*-5.79 mol% 4HB) (Fig. 5). In terms of P3HB4HB production titer, *pta*-*ackA* overexpression resulted in 1.71 g/L, significantly higher than 0.85 g/L of the control strain without *pta*-*ackA* overexpression. These results were consistent with previous P3HB producing studies, and it appears that the *pta*-*ackA* plasmid carrying recombinants were more productive than the strains without *pta*-*ackA* overexpression.

### Influence of TCA cycle intermediates on P3HB4HB synthesis

Previously, the addition of TCA cycle intermediates including α-ketoglutarate and citrate were found to be effective for increasing metabolic flux into 4HB precursor when glucose was employed as carbon source [24]. Therefore, shake flask cultures were performed to evaluate the effects of α-ketoglutarate and citrate addition on cell growth and P3HB4HB accumulation profiles in case of acetate as a main carbon source. As shown in Fig. 5, when 1 g/L of α-ketoglutarate was added, the recombinant *E. coli* reached 3.63 g/L CDW, containing 54.97 wt% P(3HB-*co*-5.80 mol% 4HB). Similarly, the addition of 1 g/L citrate as joint carbon source resulted in 3.62 g/L CDW, containing 59.48 wt% P(3HB-*co*-3.43 mol% 4HB). The addition of α-ketoglutarate and citrate as additional carbon sources increased the cell growth and P3HB4HB production, but had no obvious effect on improving 4HB monomer content.

When *E. coli* metabolizes acetate as a sole carbon and energy source, the genes involved in acetate uptake, glyoxylate cycle, TCA cycle, and gluconeogenesis were all up-regulated [12]. The addition of α-ketoglutarate or citrate could improve the supply of TCA cycle intermediates, thus may benefit cell growth and acetate assimilation. However, on this occasion, 4HB monomer content was not increased, indicating the metabolic flux towards 4HB precursor synthesis was still limited in spite of improved TCA cycle intermediate supply.

### Production of PHBV from acetate and propionate

**Fig. 6:**
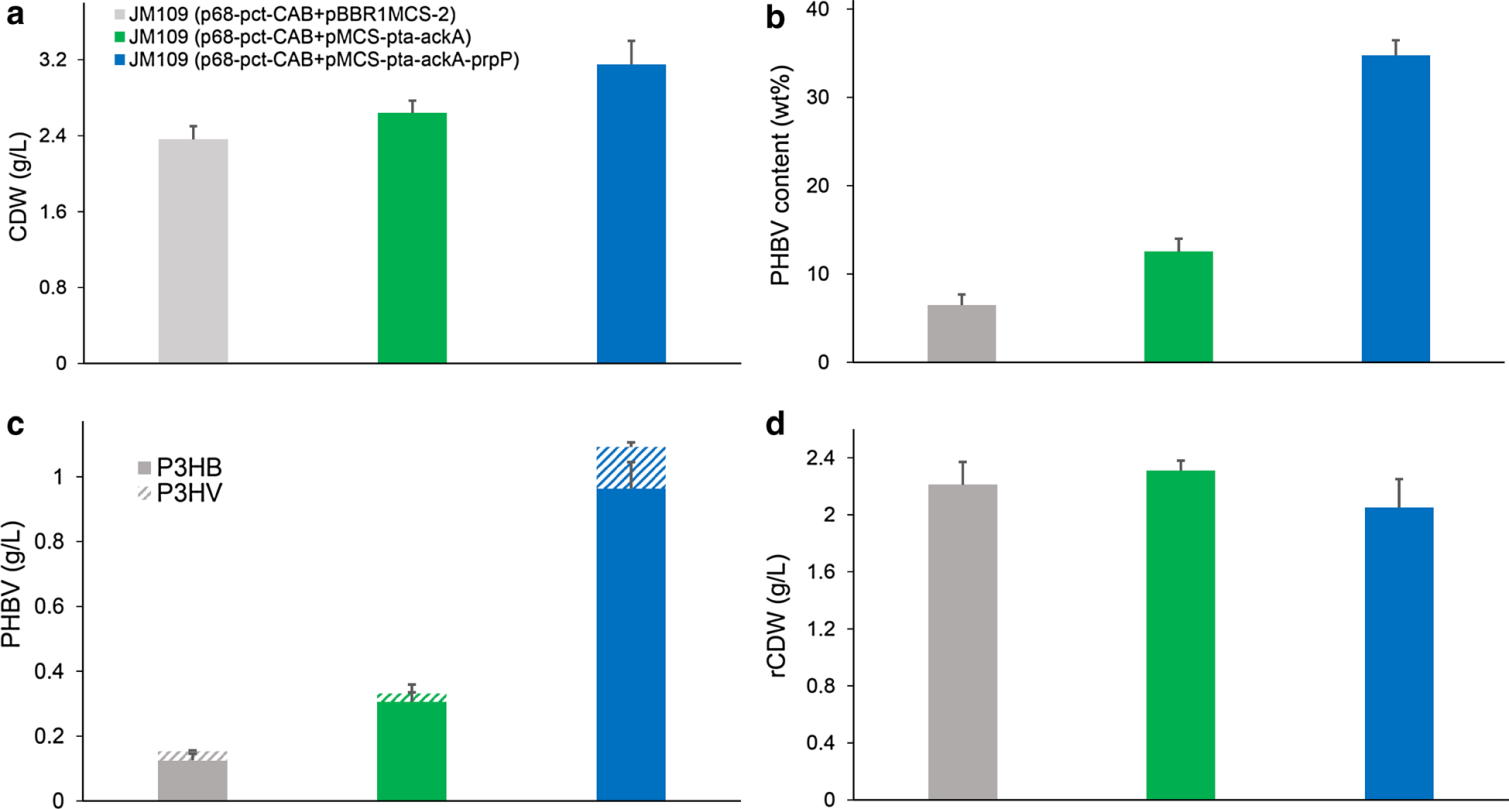
PHBV production by *E. coli* strains grown in shake flasks. *E. coli* recombinants harboring different plasmids were cultivated in MM medium supplemented with 10 g/L yeast extract, 5 g/L acetate, and 1.5 g/L propionate at 37 °C for 48 h. CDW (**a**), PHBV content (**b**), PHBV titer (**c**), and rCDW (**d**) were measured. The columns represent the averages of triplicate experiments, and the error bars represent standard deviation

Poly(3-hydroxybutyrate-*co*-3-hydroxyvalerate) (PHBV) is more flexible and tougher than P3HB homopolymer and has been commercially produced for many years [7]. The normal PHBV synthesis process requires the addition of propionate as assistant carbon source to generate propionyl-CoA, which was the precursor of 3-hydroxyvalerate (3HV) monomer (Fig. 1) [38]. The conversion of propionate to propionyl-CoA could be catalyzed by the ATP-dependent propionyl-CoA synthetase or propionyl-CoA transferase (Pct). When acetate was employed as carbon source, the intracellular pool size of acetyl-CoA should be favorable for transferring a CoA group from acetyl-CoA to propionate. To study the possibility of PHBV synthesis from acetate and propionate, plasmid p68-pct-CAB, harboring *Megasphaera elsdenii* propionyl-CoA transferase and P3HB synthesis operon *phaCAB*, was co-transformed with pMCS-pta-ackA into *E. coli* JM109. The resulting recombinant reached 2.64 g/L CDW, containing 12.52 wt% P(3HB-*co*-6.58 mol% 3HV) (Fig. 6). In contrast, the control strain JM109 (p68-pct-CAB +pBBR1MCS-2) only reached 2.36 g/L CDW containing 6.48 wt% P(3HB-*co*-15.80 mol% 3HV) under the same culture condition. PHBV production titer was increased from 0.15 to 0.33 g/L with *pta*-*ackA* overexpression, further demonstrating that *pta*-*ackA* overexpression was an effective strategy for improving acetate-dependent biopolymer synthesis.

Previously, recombinant *E. coli* harboring *phaCAB* operon and propionate permease (or propionyl-CoA synthase) failed to incorporate 3HV into the biopolymer in M9 medium containing 2 g/L yeast extract, 1 g/L sodium propionate, and 20 g/L sodium acetate [39]. However, a high concentration of yeast extract (10 g/L) was used in this study. It was speculated that a high concentration of yeast extract favors cell growth. In addition, propionyl-CoA transferase used acetyl-CoA and propionate to produce propionyl-CoA, yielding the precursor for 3HV incorporation. Therefore, our engineering strategy suggest that PHBV could be produced from acetate and propionate, yet the biopolymer content was lower than that achieved for P3HB or P3HB4HB.

Next, *prpP* gene encoding propionate permease of *R. eutropha* H16 was overexpressed to study its effect on PHBV synthesis. *E. coli* JM109 (p68-pct-CAB + pMCS-pta-ackA-prpP) resulted in 3.15 g/L CDW, containing 34.77 wt% P(3HB-*co*-10.37 mol% 3HV) (Fig. 6). The overexpression of propionate permease improved PHBV production titer from 0.33 to 1.09 g/L. Meanwhile, 3HV monomer content was increased from 6.58 to 10.37 mol%. Similar phenomenon were also observed in previous studies, in which overexpression of PrpP not only increased the 3HV monomer content but also promoted the biopolymer accumulation [40]. Propionate permease is responsible for the uptake of propionate and PrpP overexpression may improve the pool of intracellular propionate for propionyl-CoA formation. Thus, it was reasonable to find that 3HV fraction in PHBV was increased with PrpP overexpression. However, the reason for improved biopolymer production caused by PrpP overexpression was not clear and need to be discussed further.

### Use of corn steep liquor as an economical nitrogen source

Yeast extract was proved to be a critical nutritional supplement for improved PHA production (Fig. 4). To reduce the feedstock cost, many studies tried to develop cheaper supplements which can be used as an alternative or in combination with yeast extract. Corn steep liquor, a low-cost byproduct of the corn wet-milling process, contains a large amount of amino acids, peptides and vitamins [41]. Therefore, there have been increasing studies on the utilization of corn steep liquor as a nitrogen source for microbial fermentations [42, 43].

**Fig. 7:**
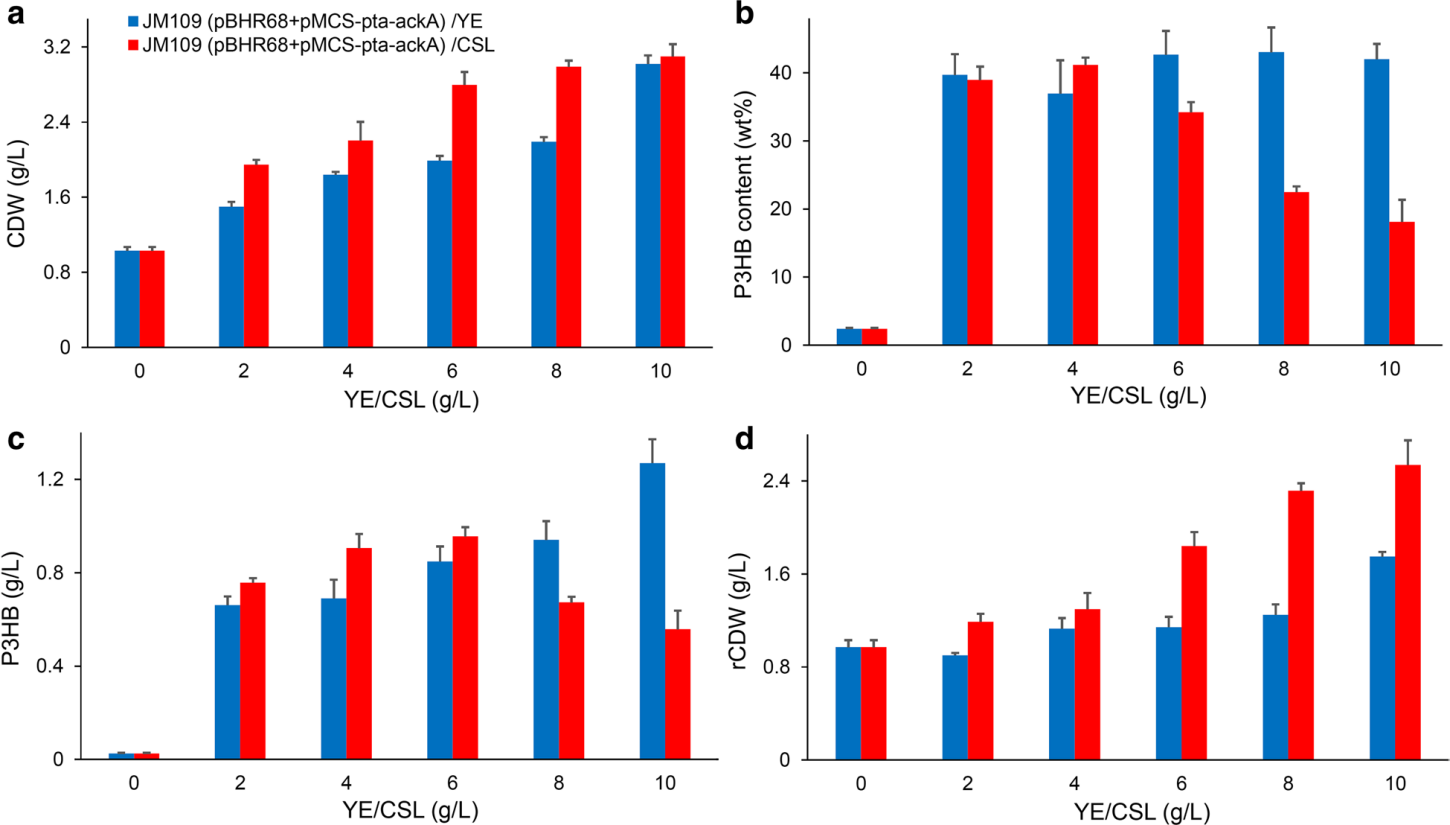
Comparative cell growth and P3HB production of engineered *E. coli* cultivated with yeast extract or corn steep liquor as nitrogen source. *E. coli* JM109 (pBHR68 + pMCS-pta-ackA) was cultivated in MM medium supplemented with 5 g/L acetate at 37 °C for 48 h. Different concentration (0, 2, 4, 6, 8, 10 g/L) of yeast extract (YE) or corn steep liquor (CSL) was added to the culture as nitrogen source. CDW (**a**), P3HB content (**b**), P3HB titer (**c**), and rCDW (**d**) were measured. The columns represent the averages of triplicate experiments, and the error bars represent standard deviation

Shake flask cultivations using various concentration (2, 4, 6, 8, 10 g/L) of corn steep liquor instead of yeast extract were performed (Fig. 7). The CDW of *E. coli* JM109 (pBHR68 + pMCS-pta-ackA) increased gradually with the increase of corn steep liquor concentration, yet the P3HB production exhibit a trend of first increasing then decreasing. The addition of 4 g/L and 6 g/L of corn steep liquor to MM medium containing 5 g/L acetate yielded 0.91 and 0.96 g/L P3HB, respectively. Although 10 g/L corn steep liquor addition led to highest CDW among the different corn steep liquor concentration condition, the P3HB concentration was only 0.56 g/L. Therefore, the favorable corn steep liquor concentration for P3HB production was 4–6 g/L under our culture condition. These results indicated that P3HB can be synthesized when corn steep liquor is employed as a nitrogen source, albeit with somewhat lower efficiency in comparison with yeast extract. The use of corn steep liquor for PHA production from acetate therefore merits further research.

In addition, we performed shake flask studies without acetate addition to evaluate the contribution of CSL to P3HB production. As shown in Additional file 1: Table S2, *E. coli* JM109 (pBHR68 + pMCS-pta-ackA) showed the highest P3HB accumulation of 0.04 g/L among all strains studied when cultivated in MM medium supplemented with 6 g/L CSL. The P3HB titer obtained without acetate was much lower than that achieved by acetate supplementation. These results further demonstrated that acetate was served as the main carbon source to support effective P3HB synthesis.

## Conclusions

In this study, *E. coli* was engineered to synthesize P3HB, P3HB4HB, and PHBV using acetate as a main carbon source. The production of biopolymers was significantly improved by the overexpression of phosphotransacetylase and acetate kinase. In shake flask cultures, the engineered *E. coli* produced 1.27 g/L of P3HB and 1.71 g/L P(3HB-*co*-5.79 mol% 4HB), respectively, with minimal medium supplemented 10 g/L yeast extract and 5 g/L acetate. When 1 g/L citrate was added as assistant carbon source, P3HB4HB production titer was increased to 2.15 g/L. The overexpression of propionyl-CoA transferase and propionate permease lead to a production titer of 1.09 g/L P(3HB-*co*-10.37 mol% 3HV) when 5 g/L acetate and 1.5 g/L propionate were simultaneously supplied. To our knowledge, this is the first study reporting the efficient production of P3HB, P3HB4HB, and PHBV using acetate as a main carbon source by engineered *E. coli*.

## Additional file

**Additional file 1: Table S1.** Oligonucleotides used in this study. **Table S2.** P3HB production by *E. coli* strains cultivated in MM medium supplemented with CSL. **Figure S1.** Effect of acetate concentration on cell growth and P3HB production.

## Abbreviations

PHA: polyhydroxyalkanoates
P3HB: poly-3-hydroxybutyrate
P3HB4HB: poly(3-hydroxybutyrate-*co*-4-hydroxybutyrate)
PHBV: poly(3-hydroxybutyrate-*co*-3-hydroxyvalerate)
3HB: 3-hydroxybutyrate
4HB: 4-hydroxybutyrate
3HV: 3-hydroxyvalerate
LB: Luria–Bertani
TB: terrific broth
MM: minimal medium
YE: yeast extract
CSL: corn steep liquor
A: acetate
KG: α-ketoglutarate
C: citrate
CDW: cell dry weight
rCDW: residual cell dry weight
GC: gas chromatography
HPLC: high-performance liquid chromatography
