# Supplementary material for: Metabolic engineering of Escherichia coli for the synthesis of polyhydroxyalkanoates using acetate as a main carbon source
Source: Microb Cell Fact. 2018 Jul 3;17:102. doi: 10.1186/s12934-018-0949-0 (PMC6029019; doi:10.1186/s12934-018-0949-0)
Supplement: Supplementary file 1 — Additional file 1: Table S1. Oligonucleotides used in this study. Table S2. P3HB production by E. coli strains cultivated in MM medium supplemented with CSL. Figure S1. Effect of acetate concentration on cell growth and P3HB production. [file 12934_2018_949_MOESM1_ESM.docx]

**Additional file 1**

Table S1 Oligonucleotides used in this study

| Primers | | Sequence |
| --- | --- | --- |
| pa1F | 5’-AAGCTTAAGAAGGAGATATACCATGTCGAGTAAGTTAGTACTGGTTC | |
| pa1R | 5’-TCTAGATTACTGCTGCTGTGCAGACTGAA | |
| pa2F | 5’-GCTAGCCGCTCATGATCGCGGCAT | |
| pa2R | 5’-CTCGAGACAGGAAGAGTTTGTAGAAACGCA | |
| acsF | 5’-AAGCTTAAGAAGGAGATATACCATGAGCCAAATTCACAAACACAC | |
| acsR | 5’-TCTAGATTACGATGGCATCGCGATAGCCT | |
| prpPF | 5’-GGATCCAAAAAGAAGGAGATATACCatgtcgtttctgatcgtgctg | |
| prpPR | 5’-AAGCTTtcagaccagtccggtcagaaaat | |

Restriction endonuclease digestion sites were underlined.

Table S2 P3HB production by *E. coli* strains cultivated in MM medium supplemented with CSL

| Plasmids | CDW (g/L) | P3HB content (wt%) | P3HB (g/L) |
| --- | --- | --- | --- |
| pBHR68 | 0.77±0.03 | 3.03±0.16 | 0.02 |
| pBHR68+pBBR1MCS-2 | 0.81±0.02 | 2.96±0.23 | 0.02 |
| pBHR68+pMCS-pta-ackA | 0.84±0.01 | 4.70±0.59 | 0.04 |

*E. coli* JM109 recombinants harboring different plasmids were cultivated in MM medium supplemented with 6 g/L CSL at 37 °C for 48 h. Data are expressed as averages and standard deviations of three parallel experiments (errors less than 0.01 not specified).

Figure S1 Effect of acetate concentration on cell growth and P3HB production

*
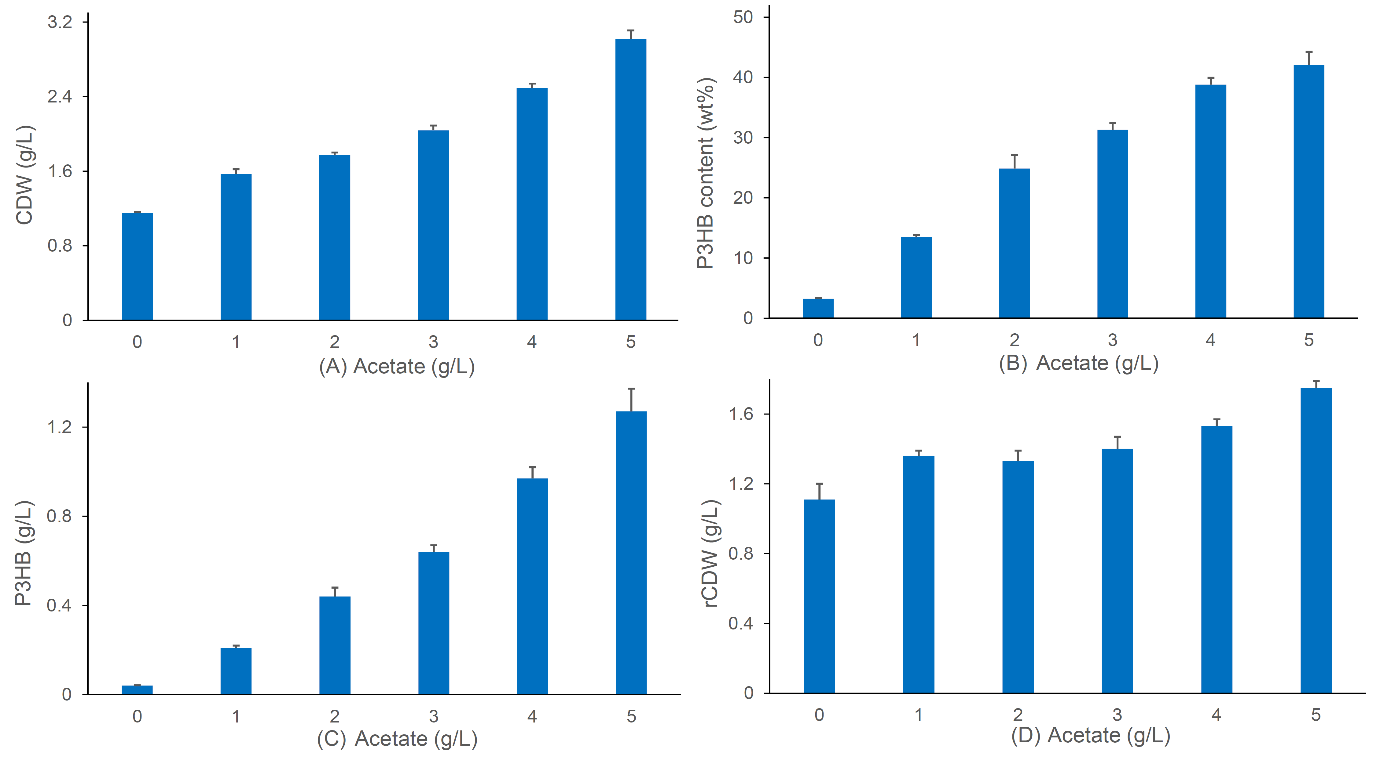
*

*E. coli* JM109 (pBHR68+pMCS-pta-ackA) was cultivated in MM medium supplemented with 10 g/L yeast extract at 37 °C for 48 h. Different concentration (0, 1, 2, 3, 4, 5 g/L) of acetate was added to the culture. The columns represent the averages of triplicate experiments, and the error bars represent standard deviation.
